# Prognostic impact of tumor-specific insulin-like growth factor binding protein 7 (IGFBP7) levels in breast cancer: a prospective cohort study

**DOI:** 10.1093/carcin/bgab090

**Published:** 2021-10-04

**Authors:** Christopher Godina, Somayeh Khazaei, Helga Tryggvadottir, Edward Visse, Björn Nodin, Karin Jirström, Signe Borgquist, Ana Bosch, Karolin Isaksson, Helena Jernström

**Affiliations:** 1 Division of Oncology, Department of Clinical Sciences, Lund, Lund University and Skåne University Hospital, Barngatan 4, SE 221 85 Lund, Sweden; 2 Department of Hematology, Oncology and Radiation Physics, Skåne University Hospital, Lund, Sweden; 3 Division of Neurosurgery, Department of Clinical Sciences, Lund, Lund University and Skåne University Hospital, Lund, Sweden; 4 Division of Oncology and Therapeutic Pathology, Department of Clinical Sciences, Lund, Lund University and Skåne University Hospital, Lund, Sweden; 5 Department of Oncology, Aarhus University and Aarhus University Hospital, Aarhus, Denmark; 6 Division of Surgery, Department of Clinical Sciences, Lund, Lund University, Lund, Sweden; 7 Kristianstad Hospital, Kristianstad, Sweden

## Abstract

The prognostic impact of insulin-like growth factor binding protein 7 (IGFBP7) in breast cancer is unclear. Host factors, including lifestyle, anthropometry and metabolic profile, might influence tumor-specific IGFBP7. This study aimed to investigate whether IGFBP7 levels and messenger ribonucleic acid (mRNA) expression are associated with the patient and tumor characteristics and prognosis in breast cancer. Patients with primary breast cancer in Lund, Sweden, were included preoperatively in the study between 2002 and 2012 (*n* = 1018). Tumor-specific IGFBP7 protein levels were evaluated with immunohistochemistry using tissue microarrays in tumors from 878 patients. IGFBP7 mRNA expression and its corresponding clinical data were obtained from The Cancer Genome Atlas and analyzed for 809 patients. Tumor-specific IGFBP7 protein levels were categorized based on Histo 300 scores into IGFBP7^low^ (6.2%), IGFBP7^intermediate^ (75.7%) and IGFBP7^high^ (18.1%). Both low IGFBP7 protein levels and mRNA expression were associated with less aggressive tumor characteristics. Overall, IGFBP7^low^ conferred low recurrence risk. The prognostic impact of IGFBP7^high^ varied according to any alcohol consumption and tamoxifen treatment. IGFBP7^high^ was associated with low recurrence risk in alcohol consumers but high recurrence risk in alcohol abstainers (*P*_interaction_= 0.039). Moreover, the combination of IGFBP7^high^ and estrogen receptor-positive tumors was associated with low recurrence risk only in tamoxifen-treated patients (*P*_interaction_= 0.029). To conclude, IGFBP7^low^ might be a good, independent prognosticator in breast cancer. The prognostic impact of IGFBP7^high^ depends on host factors and treatment. IGFBP7 merits further investigation to confirm whether it could be a suitable biomarker for treatment selection.

## Teaser

We investigated the prognostic impact of tumor-specific IGFBP7 levels in breast cancer. Overall, low IGFBP7 conferred good prognosis, while high IGFBP7 in ER^+^ tumors conferred good prognosis in tamoxifen-treated patients. IGFBP7 merits further investigation as a biomarker for treatment selection.

## Graphical Abstract

**Figure:**
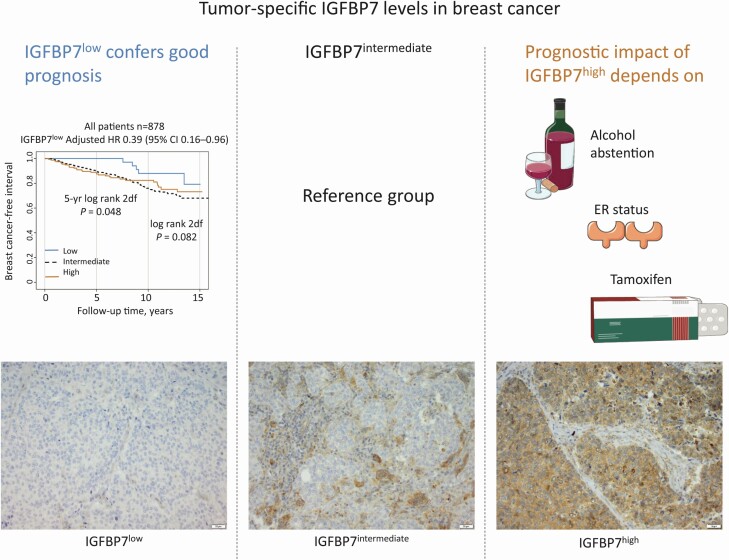

## Introduction

Breast cancer is the most common cancer among women globally (1). New tumor markers with independent prognostic and predictive value are warranted since many patients are either over- or under-treated, signaling the need for more individualized treatment (2). Dysregulation of proteins in the insulin-like growth factor (IGF)/insulin signaling pathway has been described as a driver of breast cancer initiation and progression (3). However, there is still a lack of suitable biomarkers for treatments targeting this pathway (3). An interesting candidate to be investigated as a novel biomarker is the insulin-like growth factor binding protein 7 (IGFBP7), also known as mac25/IGFBPrp1/angiomodulin (AGM)/prostacyclin-stimulating factor (PSF) or tumor adhesion factor (TAF) (4).

Interestingly, high IGFBP7 serum levels have been linked to several factors that influence breast cancer risk, such as insulin resistance, obesity and increased risk of metabolic syndrome (5,6), which may increase the risk of type 2 diabetes mellitus. Furthermore, alcohol consumption, an established risk factor for breast cancer, is also associated with a decrease of IGF-I and an increase of IGFBPs in serum (7), possibly mediated by alcohol-induced liver damage. Previous reports from breast cancer cohorts have shown that alcohol consumption was associated with improved prognosis, while high body mass index (BMI) was associated with worse prognosis (8–10).

IGFBP7 is a protein involved in the IGF/insulin signaling pathway, which plays a central role in tumorigenesis, including cell proliferation, apoptosis, migration and epithelial-mesenchymal transition (EMT) (4,11,12). There is also crosstalk between IGF/insulin and estrogen receptor (ER) pathways contributing to tumor progression and endocrine resistance (13). IGFBP7 functions by binding to the insulin or the IGF-I receptor (IGFIR), which blocks downstream signaling, hindering cell growth, survival and mitogenic activity (4,14). IGFBP7 also acts independently of the IGF/insulin pathway modulating cell growth (15). Similar to insulin-like growth factor binding protein 3 (IGFBP3), IGFBP7 can either act as a tumor promoter or suppressor in many different cancer types, including breast cancer, but the reason for this dual-action remains to be elucidated (4). To investigate whether IGFBP7 acts as a tumor suppressor or tumor promoter in breast cancer, it would be interesting to investigate both high and low (or no) IGFBP7 expression compared to intermediate expression.

A previous small Swedish study reported associations between low IGFBP7 levels and more aggressive tumor characteristics (16). However, the role of IGFBP7 in breast cancer is unclear, and conflicting *in vivo* and *in vitro* data have been published (4,14,16–21). More knowledge is needed concerning the interplay between IGFBP7, patient and tumor characteristics and prognosis. We hypothesize that host factors impact tumor-specific IGFBP7 levels and that IGFBP7 is associated with tumor characteristics. This study aimed to investigate whether IGFBP7 levels were associated with the patient and tumor characteristics and prognosis in breast cancer. Also, we investigate whether IGFBP7 messenger ribonucleic acid (mRNA) expression is associated with the patient and tumor characteristics and other proteins in the IGF/insulin-signaling pathway.

## Materials and methods

### Cohort description

**Figure 1.**
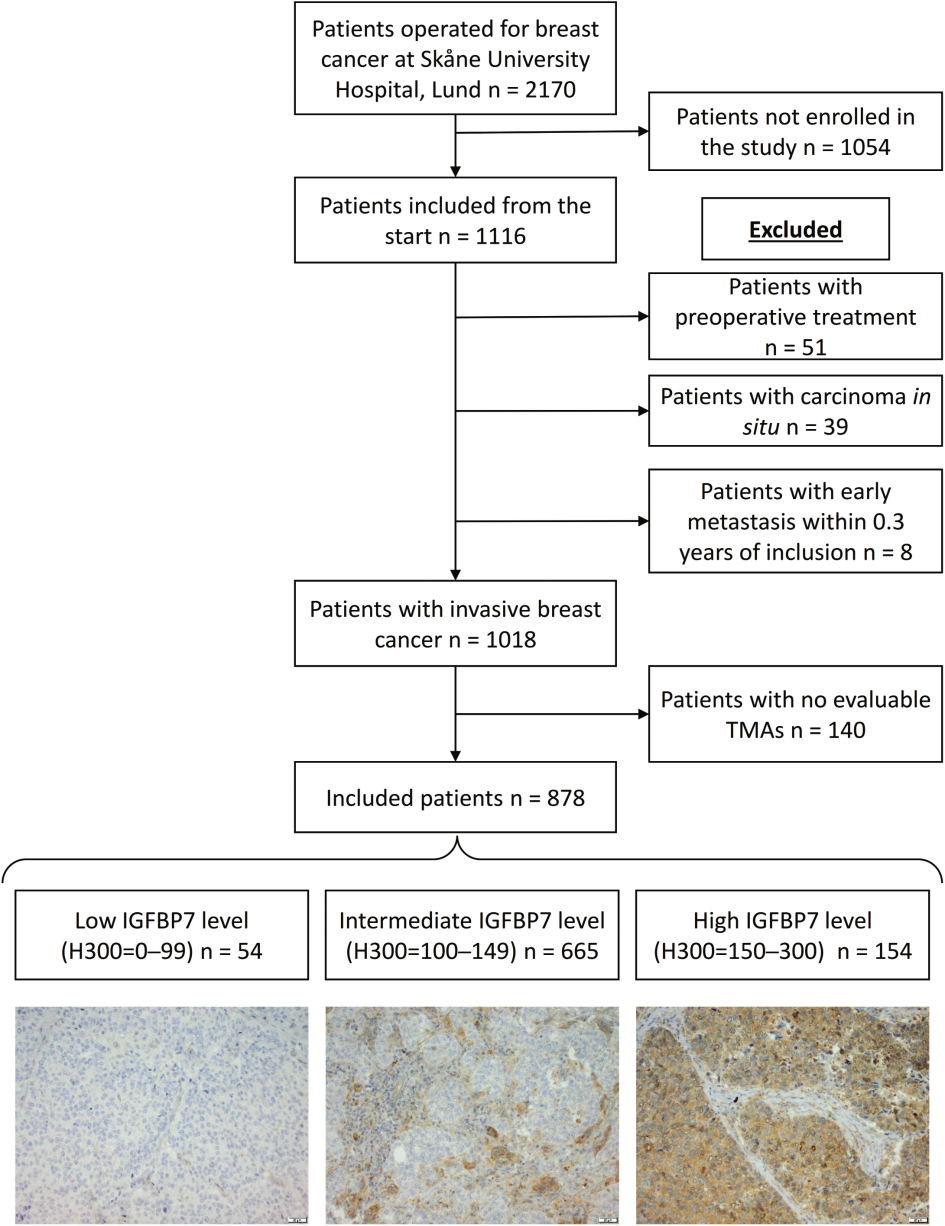
Flowchart of included and excluded patients and representative pictures of each IGFBP7 level category based on H300 index.

The BC Blood Study is a prospective population-based cohort consisting of 1116 patients operated for first primary breast cancer at Skåne University Hospital in Lund between October 2002 and June 2012. The study has ethical approval by the Lund University ethics committee (Dnr 75-02, Dnr 37-08 and amendments). All participants provided written informed consent. Patients diagnosed with other types of cancer within 10 years before their breast cancer diagnosis were not included. The participants were included after receiving their breast cancer diagnosis but before surgery. A total of 2170 patients were operated on for breast cancer at Skåne University Hospital, Lund, during the same period (22). Tissue microarrays (TMAs) were available for patients included between October 2002 and June 30, 2012. Patients with preoperative treatment (e.g. interstitial laser thermotherapy or neoadjuvant therapy), *in situ* carcinoma, and distant metastasis within 0.3 years of inclusion were excluded, leaving 1018 patients (Figure 1).

The cohort has been described in detail elsewhere (22,23). To summarize, the patients answered a questionnaire including reproductive and lifestyle factors. Research nurses took anthropometric measurements at study inclusion. Clinical data were obtained from medical records. In line with Swedish guidelines, tumors were considered ER^+^ and progesterone receptor-positive (PR^+^) if >10% of nuclei stained positive by immunohistochemistry (IHC) (8), as previously described. If the pathology reports lacked data on human epidermal growth factor receptor 2 (HER2) amplification, HER2 status was obtained from dual gene protein staining of HER2 on TMAs from the patients’ tumors. This method showed an agreement of 97.7% with available pathological assessment (24). The patients were followed up until June 30, 2019.

### Tissue microarray construction, staining and evaluation

The TMA blocks were assembled with a semi-automated tissue array device (Beecher Instruments, Sun Prairie, WI). Duplicate 1-mm cores were taken from representative non-necrotic areas with invasive tumor from archival formalin-fixed paraffin-embedded tissue from the surgical specimens. Representative regions were selected by a pathologist (K.J.) based on the re-evaluation of hematoxylin and eosin-stained slides.

TMA slides (4 µm) were deparaffinized and pretreated using the PT Link system (Agilent Technologies, Santa Clara, CA, USA). IGFBP7 staining of the TMA slides was performed using the Autostainer Plus with the EnVision FLEX high-pH kit, according to the manufacturer’s instructions (Agilent Technologies) with a polyclonal rabbit IGFBP7 antibody (ab74169, Abcam, Cambridge, UK; diluted 1:600). The antibody binds to its predicted position (29 kDa) on the western blot and was validated with short hairpin ribonucleic acid (shRNA) (25,26).

IGFBP7 protein levels were only evaluated in invasive cancer cells, and tumor cores from 878 patients were evaluable. The cytoplasmic IGFBP7 staining was evaluated by two examiners (C.G. and S.K.) blinded to tumor characteristics and patient information. Duplicate cores were evaluated jointly. The cytoplasmic intensity of IGFBP7 staining was scored as negative (0), weak (1+), moderate (2+) or strong (3+), and the estimated fraction of each intensity was recorded (0–100%). In case of discrepancy in intensity or if the estimated fraction differed by more than 10%, the cores were re-evaluated until consensus was reached. If the two examiners were unable to reach a consensus, a third evaluator (B.N.) was consulted. Blood vessels were considered positive controls, see Supplementary Figure 1 (available at *Carcinogenesis* Online) for additional histopathological images of IGFBP7 levels in various structures with the corresponding cores stained for hematoxylin and eosin. Because the IGFBP7 staining was heterogeneous, a Histo 300 (H300) index was used, whereby the intensity (0–3+) of cells was multiplied with its fraction (0–100%). This resulted in an H300 index ranging between 0 and 300 (27). The IGFBP7 levels were divided into three categories for statistical analyses based on the H300 index: IGFBP7^low^ (0–99), which corresponds to an average intensity of less than 1+ or equivalent, IGFBP7^intermediate^ (100–149) and IGFBP7^high^ (150–300), which corresponds to at least 50% of the invasive cells with an intensity of 3+ or equivalent. Representative pictures for each category are presented in Figure 1. This study followed the reporting recommendations for tumor marker prognostic study (REMARK).

### The Cancer Genome Atlas dataset

No data were available on IGFBP7 protein levels in The Cancer Genome Atlas (TCGA) database. Therefore, only mRNA expression data (pre-processed, normalized, level 3) was used. Gene-level RNA-sequence data for IGFBP7 and its corresponding clinical data and gene-level RNA-sequence data for other proteins involved in IGF/insulin signaling pathway were obtained using TCGAbiolinks (v2.18.0) package in R (v4.0.2) from the public TCGA portal https://portal.gdc.cancer.gov. The RNA-sequence data were log-transformed to reduce the ‘right skewness’ of the data. A total of 1102 samples were included from the start and were filtered to only include female primary breast cancer patients without neoadjuvant treatment and no duplicates. After filtering, 809 patients remained (Supplementary Figure 2, available at *Carcinogenesis* Online).

### Statistical analysis

For statistical analysis, SPSS^®^ software version 26 (IBM Corp, Armonk, NY, USA) and R v4.0.2 were used. Differences in clinicopathological characteristics between patients with evaluated (*n* = 878) and non-evaluated (*n* = 140) tumor-specific IGFBP7 levels were analyzed with chi-square test for categorical variables and Mann-Whitney *U*-test for continuous variables (since the values for several variables were not normally distributed). The three categories of tumor-specific IGFBP7 levels were analyzed in relation to patient and tumor characteristics with chi-square test and linear-by-linear test for categorical variables and Kruskal-Wallis or Jonckheere-Terpstra test for continuous variables.

The following variables were categorized: age at inclusion (≥50 years), coffee consumption (≥2 cups/day), alcohol abstention, preoperative smoking, ever oral contraceptive use, ever menopausal hormone therapy (MHT) use, number of children (0, 1–2, 3+), tumor size (>20 mm or muscular or skin involvement), any axillary lymph node involvement, ER^+^, PR^+^, combined ER and PR status (ER^+^PR^+^, ER^+^PR^−^, ER^−^PR^−^, ER^−^PR^+^), HER2 amplification, triple negative breast cancer (TNBC), main histological type [Ductal (or no special type), lobular, other or mixed], histological grade (I, II, III) and screening detection (in patients ages 45–74 years). Additionally, the following continuous variables were analyzed: age at inclusion (years), BMI (kg/m^2^), waist circumference (cm), waist-to-hip ratio (WHR), breast volume (ml) and age at the first child (years). Cutoffs for anthropometric features were set at BMI ≥25 kg/m^2^, WHR >0.85, waist circumference ≥80 cm (28,29) and total breast volume ≥850 ml (23).

Bilateral invasive tumors were found in 18 patients, of which eight patients had evaluable IGFBP7 levels from both tumors. For the two patients with bilateral tumors where the IGFBP7 levels based on the H300 index differed, the highest level was used. All tumor characteristics were taken from the corresponding side. Since only two cases differed, no sensitivity analysis was performed.

Kaplan-Meier and Cox proportional hazards models were used for univariable and multivariable survival analyses. Three different endpoints were used; any first breast cancer recurrence (invasive or *in situ*, locoregional, contralateral or distant metastasis), distant metastasis and death. Breast cancer-free interval (BCFI) was defined as the time from inclusion until first breast cancer recurrence. Distant metastasis-free interval (DMFI) was defined as the time from inclusion until first distant metastasis. Patients without recurrences were censored at the time of the last follow-up before emigration, death or last follow-up before July 1, 2019. Overall survival (OS) was defined as the time until death or last follow-up before July 1, 2019. For patients living in Sweden, information on subsequent death was obtained from the Swedish Population Registry.

Univariable analyses of the three different endpoints were performed with Kaplan-Meier estimates and log-rank test for the 5-year, 10-year and entire follow-up time. Crude and adjusted hazard ratios (HR) with 95% confidence interval (CI) in three different models were obtained for the three endpoints for the 5-year, 10-year and entire follow-up period. Model 1 was adjusted for age at inclusion (continuous), invasive tumor size (>20 mm or muscular or skin involvement), any axillary lymph node involvement, histological grade (III) and ER^+^. Model 2 included model 1 and was further adjusted for BMI (≥ 25 kg/m^2^), MHT use and alcohol abstention. Model 3 included model 2 and was further adjusted for chemotherapy, radiotherapy, tamoxifen and aromatase inhibitors. No adjustment was made for HER2 status in the models due to missing data for 29 patients.

To investigate effect modifications, formal two-way interaction analyses were performed between IGFBP7 categories ‘IGFBP7^low^’ or ‘IGFBP7^high^’ (with ‘IGFBP7^intermediate^’ as reference) in model 1 for the 5-year, 10-year and entire follow-up and age (≥50 years), BMI (≥25 kg/m^2^), breast volume (≥850 ml), WHR (>0.85), waist circumference (≥80 cm), coffee consumption (≥2 cups/day), alcohol abstention, preoperative smoking, ever oral contraceptive use, MHT use, tumor size (>20 mm or muscular or skin involvement), any axillary lymph node involvement, histological grade (III), ER^+^, PR^+^, HER2 amplification, TNBC, no special type (formerly ductal), lobular type, chemotherapy and radiotherapy. Additionally, among patients with ER^+^ tumors, formal two-way interaction analyses were performed in model 1 between IGFBP7 categories ‘IGFBP7^low^’ or ‘IGFBP7^high^’ (with ‘IGFBP7^intermediate^’ as reference) for the 5-year, 10-year and entire follow-up and adjuvant therapy with tamoxifen and aromatase inhibitors.

Spearman’s rank (R_s_) correlation was used to assess correlations in the TCGA database between *IGFBP7* mRNA expression and the clinical markers *ESR1*, *PGR*, *ERBB2* and age at diagnosis. Further correlations between *IGFBP7* and *IGFI*, *IGFIR*, *INSR*, *IGFBP1*, *IGFBP2*, *IGFBP3*, *IGFBP4*, *IGFBP5* and *IGFBP6* were assessed. Differences in mean *IGFBP7* mRNA expression between self-identified racial groups [White/Caucasian, Black or African American, Asian or other (including mixed and not reported)] were analyzed with one-way analysis of variance (ANOVA) with Tukey’s post hoc test. Due to scarce follow-up (median follow-up 1.3 years for patients at risk), no survival analyses were conducted for *IGFBP7* mRNA expression.

PS Power and Sample Size Calculation program version 3.1.12 from (Vanderbilt University, Nashville, TN, USA) was used to perform power calculations. We assumed for the power calculation that with 855 patients with 18% having IGFBP7^high^ and 6% having IGFBP7^low^, a 10-year accrual time with an additional 7-year follow-up, a median follow-up of 9 years, 80% power and an α of 0.05, we would be able to detect true HRs of ≤0.731 or ≥1.415 and ≤0.618 or ≥1.764, respectively (30). *P*-values < 0.05 were considered statistically significant, and all *P*-values are two-tailed. Since this is an exploratory study, nominal *P*-values are presented without adjustment for multiple testing (31).

## Results

### Patient characteristics and IGFBP7 levels

Out of the 1018 patients included in the study, 878 (86.2%) had tumors that could be evaluated for IGFBP7 levels. Among these patients, IGFBP7 H300 ranged from 0 to 300 with a median of 110 [interquartile range (IQR) 100–130]. Fifty-four patients (6.2%) had a low (H300 0–99) IGFBP7 level, 665 (75.7%) had an intermediate (H300 100–149) level and 159 (18.1%) had a high (H300 150–300) level. See Figure 1 for representative images of staining categories. IGFBP7^low^ was weakly associated with prior MHT use (*P* = 0.025) and daily coffee consumption of ≥2 cups (*P* = 0.053). There were no other statistically significant differences in patient characteristics depending on IGFBP7 levels (Table 1).

**Table 1.** Tumor-specific IGFBP7 levels in relation to patient characteristics

	Patients with evaluable TMAs	Missing	IGFBP7^low^	IGFBP7^intermediate^	IGFBP7^high^	Patients with non-evaluable TMAs
	No. of patients (%) or median (IQR)	n	No. of patients (%) or median (IQR)	No. of patients (%) or median (IQR)	No. of patients (%) or median (IQR)	No. of patients (%) or median (IQR)
	*n* =878 (100%)		*n* = 54 (6.2%)	*n* = 665 (75.7%)	*n* = 159 (18.1%)	*n* = 140
Age at inclusion, years	61.1 (52.3–68.2)	0	61.5 (58.3–69.0)	61.1 (52.0–68.1)	60.3 (51.9–69.4)	61.1 (51.7–67.8)
Age at inclusion ≥ 50 years	708 (80.6%)	0	49 (90.7%)	535 (80.5%)	124 (78.0%)	108 (77.1%)
BMI, kg/m^2^	25.1 (22.5–28.4)	25	25.2 (22.4–28.7)	25.0 (22.4–28.2)	25.1 (22.8–29.1)	25.1 (22.5–28.2)
Waist circumference, cm	87 (79–97)	33	86 (78–96)	87 (79–97)	88 (79–97)	88 (79–95)
Waist-to-hip ratio	0.86 (0.81–0.90)	33	0.85 (0.81–0.89)	0.86 (0.81–0.91)	0.85 (0.81–0.91)	0.86 (0.81–0.90)
Breast volume, ml	1000 (650–1500)	134	975 (700–1400)	1000 (650–1500)	1000 (600–1600)	1000 (700–1600)
Alcohol abstainer, yes	98 (11.2%)	3	5 (9.3%)	75 (11.3%)	18 (11.5%)	8 (5.7%)
Preoperative smoker, yes	170 (19.4%)	2	10 (18.5%)	125 (18.8%)	35 (22.2%)	36 (25.7%)
Coffee, ≥2 cups per day	707 (80.5%)	0	50 (92.6%)	527 (79.2%)	130 (81.8%)	117 (83.6%)
Oral contraceptives, ever	619 (70.6%)	1	34 (63.0%)	475 (71.5%)	110 (69.2%)	103 (73.6%)
Hormonal intrauterine device, ever	147 (17.2%)	21	4 (8.2%)	119 (18.3%)	24 (15.3%)	27 (19.6%)
Menopausal hormone therapy, ever	388 (44.3%)	3	33 (62.3%)	286 (43.1%)	69 (43.4%)	59 (42.1%)
Age at menarche, years	13 (12–14)	2	13 (12–14)	13 (12–14)	13 (12–14)	13 (12–14)
Number of children						
0 (Nulliparous)	109 (12.4%)		8 (14.8%)	77 (11.6%)	24 (15.1%)	13 (9.3%)
1–2	526 (59.9%)		32 (59.3%)	405 (60.9%)	89 (56.0%)	102 (72.9%)
3+	243 (27.7%)		14 (25.9%)	183 (27.5%)	46 (28.9%)	25 (17.9%)
Age at first child in parous women, years	24 (21–28)	2	23 (20–27)	24 (22–28)	25 (21–28)	25 (23–29)

### Tumor characteristics and IGFBP7 levels

Higher categories of IGFBP7 levels were associated with ER^−^ status (*P*_trend_ < 0.001), PR^−^ status (*P*_trend_*<* 0.001) and lower histological grade (*P*_trend_ < 0.001; Table 2). The trend was observed only for concordant ER PR status (both *P*_trends_ < 0.001) but not for discordant ER and PR status. Additionally, IGFBP7^low^ was associated with invasive lobular-type breast cancer (*P* < 0.001). There were no other statistically significant differences in tumor characteristics depending on IGFBP7 levels (Table 2).

**Table 2.** Tumor-specific IGFBP7 levels in relation to other tumor characteristics.

	Patients with evaluable TMAs	Missing	IGFBP7^low^	IGFBP7^intermediate^	IGFBP7^high^	Patients with non-evaluable TMAs
	No. of patients (%)	*n*	No. of patients (%)	No. of patients (%)	No. of patients (%)	No. of patients (%)
	*n* =878 (100%)		*n* = 54 (6.2%)	*n* = 665 (75.7%)	*n* = 159 (18.1%)	*n* = 140
Invasive tumor size		0				
≤20 mm	633 (72.1%)		40 (74.1%)	484 (72.8%)	109 (68.6%)	109 (77.9%)
>20 mm or muscular or skin involvement	245 (27.9%)		14 (25.9%)	181 (27.2%)	50 (31.4%)	31 (22.1%)
Axillary lymph node involvement		2				
Any	341 (38.9%)		18 (33.3%)	262 (39.5%)	61 (38.6%)	48 (34.3%)
Receptor status						
ER^+^	768 (87.6%)	1	53 (98.1%)	604 (91.0%)	111 (69.8%)	126 (90.0%)
PR^+^	619 (70.6%)	1	46 (85.2%)	489 (73.6%)	84 (52.8%)	102 (72.9%)
Combined ER and PR status		1				
ER^+^ PR^+^	614 (70.0%)		46 (85.2%)	487 (73.3%)	81 (50.9%)	102 (72.9%)
ER^+^ PR^−^	154 (17.6%)		7 (13.0%)	117 (17.6%)	30 (18.9%)	24 (17.1%)
ER^−^ PR^−^	104 (11.9%)		1 (1.9%)	58 (8.7%)	45 (28.3%)	14 (10.0%)
ER^−^ PR^+^	5 (0.6%)		0 (0.0%)	2 (0.3%)	3 (1.9%)	0 (0.0%)
HER2 amplification^a^	95 (11.2%)	29	5 (10.0%)	68 (10.6%)	22 (14.2%)	14 (13.2%)
Triple negative	68 (7.8%)	3	0 (0.0%)	33 (5.0%)	35 (22.0%)	6 (4.4%)
Main histological type		0				
Ductal (or no special type)	730 (83.1%)		37 (68.5%)	553 (83.2%)	140 (88.1%)	94 (67.1%)
Lobular	91 (10.4%)		16 (29.6%)	68 (10.2%)	7 (4.4%)	26 (18.6%)
Other or mixed	57 (6.5%)		1 (1.9%)	44 (6.6%)	12 (7.5%)	20 (14.3%)
Histological grade		0				
I	212 (24.2%)		15 (27.8%)	173 (26.1%)	24 (15.1%)	45 (32.1%)
II	436 (49.7%)		33 (61.1%)	347 (52.3%)	56 (35.2%)	67 (47.9%)
III	229 (26.1%)		6 (11.1%)	144 (27.1%)	79 (47.9%)	28 (20.0%)
Mode of detection (45–74 years)						
Screening detected	482 (65.2%)	0	32 (68.1%)	374 (66.1%)	76 (60.3%)	87 (72.5%)
Ever treatment by last follow-up prior to any event						
Chemotherapy	229 (26.1%)	0	9 (16.7%)	158 (23.8%)	62 (39.0%)	30 (21.4%)
Radiotherapy	562 (64.0%)	0	30 (55.6%)	430 (64.7%)	102 (64.2%)	82 (58.6%)
HER2 amplified^a^						
Trastuzumab	59 (62.1%)	0	4 (80.0%)	42 (61.8%)	13 (59.1%)	11 (78.6%)
ER^+^ tumors						
Tamoxifen	498 (64.8%)	0	36 (67.9%)	382 (63.2%)	80 (72.1%)	74 (58.7%)
Aromatase inhibitor	326 (42.4%)	0	24 (45.3%)	257 (42.5%)	45 (40.5%)	45 (35.7%)
Type of event						
Any breast cancer event	174 (19.8%)	0	5 (9.3%)	138 (20.8%)	31 (19.5%)	21 (15.0%)
Distant metastasis	112 (12.8%)	0	3 (5.6%)	89 (13.4%)	20 (12.6%)	10 (7.1%)
Death	174 (19.8%)	0	9 (16.7%)	128 (19.2%)	37 (23.3%)	14 (13.2%)

^a^Two additional patients also received trastuzumab due to HER amplification on the contralateral side, that is, a total of 61 patients received trastuzumab.

### Patients with tumors non-evaluable for IGFBP7 staining

Patients with non-evaluable tumors for IGFBP7 were similar to patients with evaluable tumors except for alcohol abstention, number of children and age at the first child (Table 1). Only histological grade and main histological type differed between evaluable and non-evaluable tumors. There were also fewer deaths among patients with non-evaluable tumors (Table 2).

### TCGA mRNA expression

*IGFBP7* mRNA expression was positively correlated with the mRNA expression of *PGR*, *IGF1*, *IGFBP2*, *IGFBP3*, *IGFBP4*, *IGFBP5* and *IGFBP6* (all R_s_ ≥ 0.13 and *P-*values < 0.001). In contrast, *IGFBP7* mRNA expression was negatively correlated with age and *ESR1* (both R_s_ ≥ 0.074 and *P-*values ≤ 0.034). There were no statistically significant correlations between *IGFBP7* expression and *ERBB2*, *IGFIR* or *INSR*. Moreover, there was no difference in *IGFBP7* mRNA expression between self-reported racial groups (*n* = 736); White/Caucasian, Black or African American and Asian (*P* > 0.3).

### Tumor-specific IGFBP7 protein levels and prognosis

The patients in the BC Blood cohort were followed for up to 15 years, and for the 624 patients still at risk, the median follow-up time was 9.1 years (IQR 7.0–11.1 years). A total of 174 patients had a recurrence during the follow-up, of which 112 (64.4%) had distant metastasis. Also, a total of 174 patients died during the study period, and 94 (56.0%) of these patients had a breast cancer recurrence (Table 2).

**Figure 2.**
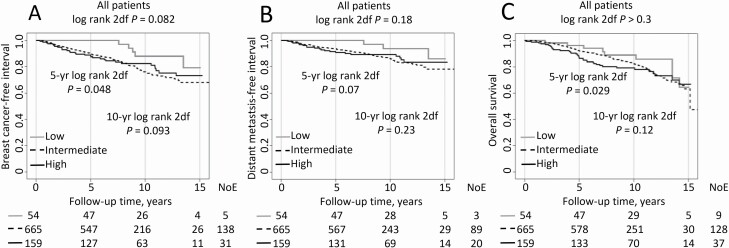
Kaplan-Meier estimates of (**A)** breast cancer-free interval, (**B**) distant metastasis-free interval and (**C**) overall survival in relation to IGFBP7 levels in all patients. The number of patients is indicated at each follow-up. The study is ongoing; thus, the number of patients decreases with each follow-up.

IGFBP7^low^ was associated with a lower 5-year recurrence risk (no event) compared to IGFBP7^intermediate^ (9.9%) and IGFBP7^high^ (11.8%). For patients with IGFBP7^low^, the first recurrence (a distant metastasis) occurred after 7.6 years. IGFBP7^low^ was associated with a lower recurrence risk also with longer follow-up times in the univariable analysis and multivariable analyses (Figure 2A and Table 3). For the multivariable analyses, IGFBP7^intermediate^ was used as reference. Similar patterns were seen for DMFI (Figure 2B and Table 3). There was no difference in OS between IGFBP7^low^ and IGFBP7^intermediate^. IGFBP7^high^ had shorter OS during the first 5 years of follow-up in the univariable but not the multivariable analyses (Figure 2C and Table 3).

**Table 3.** Multivariable Cox regression survival analyses of IGFBP7 levels in relation to recurrences, distant metastases and death due to any cause for the entire, 10-year, and 5-year follow-up period

Breast cancer recurrence										
	Total	Events	Crude		Model 1		Model 2		Model 3	
IGFBP7 levels	*n*	*n*	HR	(95% CI)	HR_adj_	(95% CI)	HR_adj_	(95% CI)	HR_adj_	(95% CI)
Low	54	5	0.38	0.16 to 0.92	0.39	0.16 to 0.96	0.43	0.17 to 1.04	0.40	0.16 to 0.99
Intermediate	665	138	Ref.		Ref.		Ref.		Ref.	
High	159	31	0.92	0.62 to 1.35	0.77	0.51 to 1.16	0.79	0.53 to 1.20	0.78	0.52 to 1.18
		*P*_trend_	>0.3		>0.3		>0.3		>0.3	
IGFBP7 levels	*n*	*n*	10-year		10-year		10-year		10-year	
Low	54	4	0.36	0.13 to 0.97	0.38	0.14 to 1.03	0.40	0.15 to 1.09	0.38	0.14 to 1.03
Intermediate	665	123	Ref.		Ref.		Ref.		Ref.	
High	159	25	0.86	0.56 to 1.32	0.70	0.45 to 1.10	0.71	0.45 to 1.11	0.70	0.44 to 1.10
		*P*_trend_	>0.3		>0.3		>0.3		>0.3	
IGFBP7 levels	*n*	*n*	5-year		5-year		5-year		5-year	
Low	54	0	No event		No event		No event		No event	
Intermediate	665	63	Ref.		Ref.		Ref.		Ref.	
High	159	18	1.21	0.72 to 2.05	0.86	0.50 to 1.50	0.84	0.48 to 1.47	0.85	0.48 to 1.51
		*P*_trend_	0.043		>0.3		>0.3		>0.3	
Distant metastasis										
	Total	Events	Crude		Model 1		Model 2		Model 3	
IGFBP7 levels	*n*	*n*	HR	(95% CI)	HR_adj_	(95% CI)	HR_adj_	(95% CI)	HR_adj_	(95% CI)
Low	54	3	0.36	0.11 to 1.13	0.36	0.11 to 1.12	0.38	0.12 to 1.12	0.34	0.10 to 1.08
Intermediate	665	89	Ref.		Ref.		Ref.		Ref.	
High	159	20	0.91	0.56 to 1.48	0.67	0.40 to 1.11	0.68	0.41 to 1.14	0.67	0.40 to 1.13
		*P*_trend_	>0.3		>0.3		>0.3		>0.3	
IGFBP7 levels	*n*	*n*	10-year		10-year		10-year		10-year	
Low	54	2	0.31	0.08 to 1.28	0.33	0.08 to 1.34	0.35	0.09 to 1.42	0.32	0.08 to 1.33
Intermediate	665	73	Ref.		Ref.		Ref.		Ref.	
High	159	16	0.92	0.54 to 1.59	0.66	0.38 to 1.15	0.66	0.37 to 1.16	0.66	0.37 to 1.16
		*P*_trend_	>0.3		>0.3		>0.3		>0.3	
IGFBP7 levels	*n*	*n*	5-year		5-year		5-year		5-year	
Low	54	0	No event		No event		No event		No event	
Intermediate	665	40	Ref.		Ref.		Ref.		Ref.	
High	159	14	1.48	0.80 to 2.71	0.94	0.50 to 1.78	0.93	0.48 to 1.80	0.93	0.48 to 1.81
		*P*_trend_	0.029		>0.3		>0.3		>0.3	
Death										
	Total	Events	Crude		Model 1		Model 2		Model 3	
IGFBP7 levels	*n*	*n*	HR_adj_	(95% CI)	HR_adj_	(95% CI)	HR_adj_	(95% CI)	HR_adj_	(95% CI)
Low	54	9	0.72	0.37 to 1.42	0.72	0.36 to 1.42	0.75	0.38 to 1.48	0.72	0.36 to 1.43
Intermediate	665	128	Ref.		Ref.		Ref.		Ref.	
High	159	37	1.13	0.78 to 1.63	0.89	0.60 to 1.31	0.85	0.56 to 1.27	0.84	0.56 to 1.27
		*P*_trend_	0.25		>0.3		>0.3		>0.3	
IGFBP7 levels	*n*	*n*	10-year		10-year		10-year		10-year	
Low	54	5	0.61	0.25 to 1.51	0.67	0.27 to 1.64	0.72	0.29 to 1.76	0.69	0.28 to 1.71
Intermediate	665	95	Ref.		Ref.		Ref.		Ref.	
High	159	31	1.40	0.93 to 2.09	1.13	0.74 to 1.74	1.08	0.69 to 1.70	1.08	0.69 to 1.71
		*P*_trend_	0.039		>0.3		>0.3		>0.3	
IGFBP7 levels	*n*	*n*	5-year		5-year		5-year		5-year	
Low	54	2	0.50	0.12 to 2.06	0.58	0.14 to 2.40	0.66	0.16 to 2.73	0.65	0.16 to 2.73
Intermediate	665	49	Ref.		Ref.		Ref.		Ref.	
High	159	21	1.82	1.09 to 3.04	1.35	0.78 to 2.34	1.32	0.74 to 2.36	1.38	0.77 to 2.48
		*P*_trend_	0.009		0.17		0.24		0.19	

Adjusted model 1: Age at inclusion, tumor size, nodal status, grade III and ER status. Missing data for four patients for at least one variable.

Adjusted model 2: Model 1+ BMI ≥25 Kg/m^2^, MHT and alcohol abstention. Missing data for 35 patients for at least one variable.

Adjusted model 3: Model 2+ chemotherapy, radiotherapy, tamoxifen and aromatase inhibitors. Missing data for 35 patients for at least one variable.

### Effect modifications between IGFBP7 levels and clinicopathological factors on prognosis

**Figure 3.**
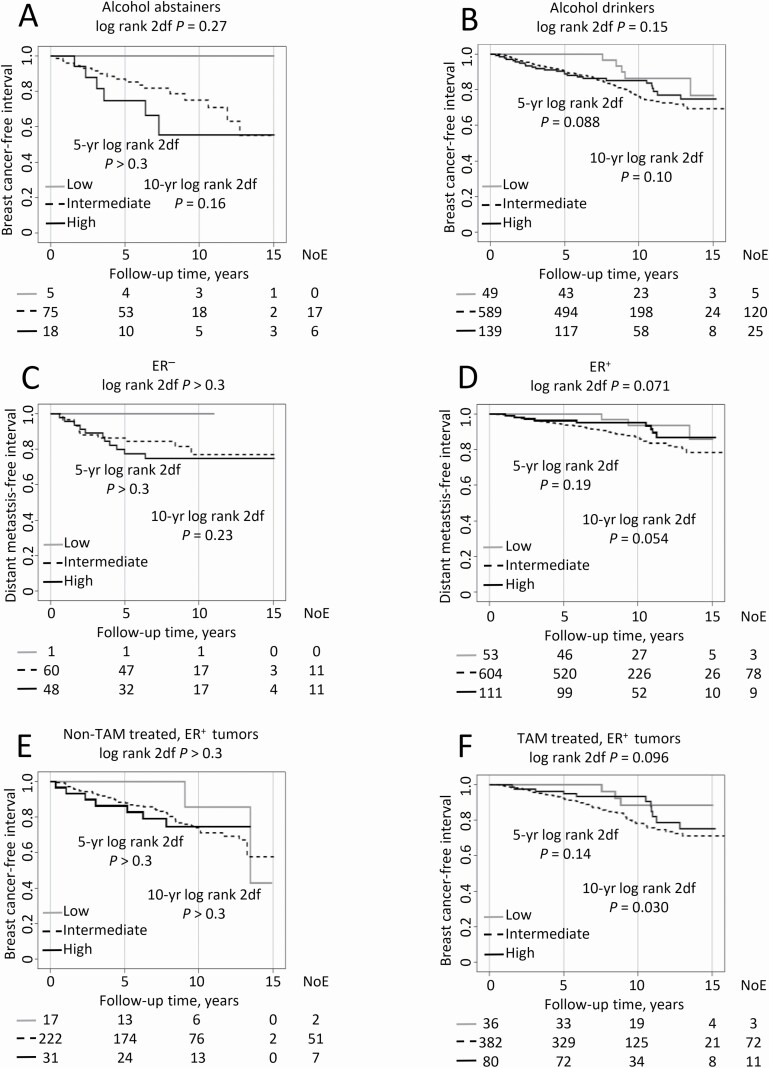
Kaplan-Meier estimates of (**A, B**) breast cancer-free interval in all patients stratified by alcohol abstention, (**C, D**) distant metastasis-free interval in all patients stratified by ER status and (**E, F**) breast cancer-free interval in patients with ER^+^ tumors stratified by ever tamoxifen treatment in relation to IGFBP7 levels. The study is ongoing; thus, the number of patients decreases with each follow-up.

There were significant interactions between IGFBP7^high^ and several clinicopathological factors with respect to prognosis using IGFBP7^intermediate^ as reference. Thus, subgroup analyses were stratified by alcohol abstention, ER status and tamoxifen treatment among patients with ER^+^ tumors. In alcohol abstainers, IGFBP7^high^ was associated with higher 10-year recurrence risk HR_adj_ 4.03 (95% CI, 1.03 to 15.74), but in alcohol drinkers, IGFBP7^high^ was associated with lower recurrence risk HR_adj_ 0.56 (95% CI, 0.34 to 0.93; *P*_interaction_ = 0.039; Figure 3A and 3B). IGFBP7^high^ was not associated with 10-year DMFI in patients with ER^−^ tumors while borderline associated with lower 10-year distant metastasis risk in patients with ER^+^ tumors HR_adj_ 0.40 (95% CI, 0.16 to 1.01; *P*_interaction_ = 0.037; Figure 3C and D). When restricting the analyses to patients with ER^+^ tumors, IGFBP7^high^ was not associated with 10-year BCFI while associated with lower 10-year recurrence risk HR_adj_ 0.35 (95% CI, 0.14 to 0.89) in tamoxifen-treated patients (*P*_interaction_ = 0.029; Figure 3E and F). There was also an interaction between IGFBP7^high^ and TNBC (*P*_interaction_ = 0.046) but no significant association between IGFBP7^high^ and OS in either subgroup.

Furthermore, there were significant interactions between IGFBP7^low^ and several factors with respect to prognosis, including tamoxifen treatment among patients with ER^+^ tumors and no special type (formerly ductal), using IGFBP7^intermediate^ as reference. IGFBP7^low^ and ER^+^ tumors were borderline associated with longer OS in tamoxifen-treated patients, HR_adj_ 0.37 (95% CI, 0.13 to 1.03), but not in non-tamoxifen-treated patients (*P*_interaction_ = 0.031). In patients with breast cancer of no special type, IGFBP7^low^ was borderline associated with longer OS HR_adj_ 0.37 (95% CI, 0.13 to 1.02; compared to other histological types for the entire and 10-year follow-up (both *P*_interactions_ ≤ 0.039; Supplementary Figure 3, available at *Carcinogenesis* Online). Also, there was an interaction between IGFBP7^low^ and lobular type on OS (*P*_interaction_ = 0.033) but no significant associations in either subgroup.

Since IGFBP7 protein levels were also associated with PR, we performed further adjustments for PR in the survival analyses. The effect estimates remained essentially the same. However, two models became significant: (i) IGFBP7^high^ was associated with lower 10-year distant metastasis risk HR_adj_ 0.39 (95% CI, 0.15 to 0.99) in patients with ER^+^ tumors. (ii) IGFBP7^low^ was associated with longer OS HR_adj_ 0.35 (95% CI, 0.13 to 0.99) in patients with breast cancer of no special type. The effect estimates remained essentially the same after adjustment for smoking, coffee and trastuzumab, respectively. In restriction analyses where only patients with known HER2 status were included, the effect estimates remained essentially the same. Similarly, in restriction analyses where patients with bilateral tumors (*n* = 18) were excluded, the effect estimates remained essentially the same.

## Discussion

The main findings were that patients with IGFBP7^low^ had less aggressive tumor characteristics, a lower risk of breast cancer recurrence, and a higher frequency of prior MHT use. Moreover, the prognostic impact of IGFBP7^high^ varied significantly according to alcohol abstention, ER status and tamoxifen treatment.

To our knowledge, no study has previously reported the finding of MHT or lobular-type breast cancer being associated with IGFBP7^low^, and it has been previously shown that MHT is associated with lobular-type cancer (32). MHT modulates the serum levels of proteins involved in the IGF system differently according to the type of MHT (33,34) and may, therefore, influence IGFBP7 levels. MHT was weakly associated with lower serum levels of IGFBP7 in a subset of patients in the same cohort (35). The IGF/insulin and ER pathways, through which MHT mainly functions, are interconnected in breast cancer (13,36). A previous study based on the same breast cancer cohort reported that MHT was associated with lobular cancer and low histological grade (37). This is in line with the present finding of IGFBP7^low^ being associated with both lobular type breast cancer and low histological grade. This association remained after stratification by MHT status. We hypothesize that the association between IGFBP7 and MHT is partly mediated via the crosstalk between ER and IGF/insulin pathways. Further, E-cadherin downregulation is hallmark of lobular-type breast cancer (38). Interestingly, mechanistic studies of colorectal cancer have shown that knockdown of IGFBP7 impacts proteins associated with EMT, such as downregulation of E-cadherin (25). Our findings of IGFBP7^low^ in lobular-type cancer and in MHT users are aligned with the results of the mechanistic study.

Both higher IGFBP7 levels and expression were associated with ER^−^, which is in disagreement with a previous smaller study (16). Interestingly, the phosphatidylinositol 3-kinase (PI3K) pathway activation is more frequently associated with ER positivity in breast cancer. This pathway is regulated by IGFIR, in which signaling activity, in turn, is modulated by IGFBP7 (4,14,38), indicating a potential pathway linking IGFBP7 and ER expression. Additionally, IGFBP7 mRNA expression was positively correlated with the expression of other proteins involved in the IGF/insulin pathway, supporting previous theories that IGFBPs are related to each other and function as a cooperative (4). The majority of the associations between IGFBP7 and tumor characteristics went in the same direction for protein and mRNA expression, except PR. This might be due to post-translation regulation of protein levels (39).

Moderate to high coffee consumption was borderline associated with IGFBP7^low^, which to our knowledge, has not previously been reported. In the same cohort, high coffee consumption was associated with lower IGF1R levels, less aggressive tumors, and improved prognosis in patients treated with radiotherapy or tamoxifen (40,41). In contrast to serum IGFBP7 levels (5,6), tumor-specific IGFBP7 levels were not associated with anthropometric features in this study. Similarly, serum IGFBP7 levels were not associated with anthropometric features in a subset of patients in the same cohort (35), suggesting that serum and tumor-specific IGFBP7 represent two or several independent functions. Similar results were observed in colorectal cancer where serum and tumor-specific IGFBP7 levels were not correlated (4).

The participants in the BC Blood cohort are considered representative of breast cancer patients operated in Skåne University Hospital in Lund and similar to the TCGA cohort (38), the BC Blood cohort includes patients of all ages (22). Approximately 300 000 people live in the catchment area, and there are no private clinics performing breast cancer surgeries. The power was adequate for the entire BC Blood cohort, and clinically relevant effect sizes were detectable. However, the subgroups of patients with IGFBP7^low^ or IGFBP7^high^ were relatively small, and the power was, therefore, lower in subgroup analyses. Survival curves were proportional for the 5-year follow-up but not the entire follow-up. HRs thus present the mean hazard over time. Since the questionnaires were answered preoperatively, the risk for recall bias and survivor bias was minimized. No adjustments were made for HER2 status and trastuzumab treatment in the main models due to missing data on several patients included before November 2005. However, in restriction analyses in patients with known HER2 status, the effect estimates remained essentially the same as for all patients whether or not trastuzumab was included in the models. Age influences the expression of several prognostic markers as well as breast cancer prognosis (2). In the TCGA cohort, age was negatively correlated with *IGFBP7* mRNA expression. However, tumor-specific IGFBP7 levels appeared stable across ages in the BC Blood cohort and all adjusted models included age as a covariate limiting the risk of age confounding our results. Body measurements were taken by research nurses, strengthening reliability. The clinical data are reliable since they were obtained from pathology reports, patient charts and population registers. Ethnicity was not denoted, but the majority of patients were of Swedish or European background. Since *IGFBP7* expression did not vary across the three major ethnic groups in the TCGA data, protein levels may also be similar across ethnic groups.

Missing tumor tissue was somewhat more common among patients who consumed alcohol. However, alcohol abstention was not associated with IGFBP7 levels. Also, the differences in tumor characteristics indicated that non-evaluated tumors were somewhat less aggressive than evaluated tumors. This might lead to an underestimation of the of IGFBP7^low^ frequency. As there is no established protocol for IGFBP7 staining, the staining methods were optimized for this study. Neither is there a standard procedure for IGFBP7 evaluation, making comparisons between different studies difficult. Landberg *et al*. (16) deemed the IGFBP7 staining on whole section slides to be homogenous enough to be evaluated with TMAs in breast cancer.

IGFBP7^high^ was not associated with less aggressive tumor characteristics and improved prognosis, contradicting the findings of a previous study (16). Instead, IGFBP7^low^ was an independent prognostic factor for low recurrence risk. It remains unclear why a tumor suppressor would be associated with more aggressive tumors and a worse prognosis. Our findings support the notion that tumor-specific IGFBP7 acts as a promoter rather than a suppressor in breast cancer. IGFBP7, similar to IGFBP3, might have a dual role as a tumor suppressor within the IGF/insulin system while simultaneously having tumor-promoting effects through other pathways (4). Alternatively, IGFBP7 levels might rise in response to more aggressive breast cancer to slow its spread. In the TCGA cohort, IGFBP7 and IGFBP3 were positively correlated and others have shown that these proteins share signaling pathways. IGFBP3 is the main binding protein of IGF-I while IGFBP7 binds insulin with high affinity and has a lower affinity for IGF-I (4). Both of these IGFBPs also have roles independent of IGF-I and insulin (4). Perhaps, further investigation into the interplay between IGFBP3 and IGFBP7 could shed more light on why IGFBP7^high^ impacts prognosis differently depending on alcohol abstention, ER status and tamoxifen use (4). However, investigation of IGFBP3 lies outside the scope of this study.

This study is the first to report effect modifications according to alcohol abstention, ER status and tamoxifen treatment. We have previously reported that alcohol abstainers are less likely to adhere to endocrine treatment in this cohort (42), suggesting that these two factors may be linked. Some effects of alcohol in breast cancer are proposed to be mediated through the IGF system (43) and might attenuate the anti-proliferative effect of IGFBP7 by decreasing serum IGF-I levels (7). Similarly, tamoxifen may decrease *IGF1* gene expression in breast cancer cells (44). Others have shown activated IGFIR to be a marker of tamoxifen resistance (45). IGFBP7 modulates IGFIR signaling (14), suggesting a synergistic anti-proliferative mechanism whereby high IGFBP7 levels counteract tamoxifen resistance. This mechanism could explain why IGFBP7^high^ was associated with better prognosis only in tamoxifen-treated patients in the present study. It has been shown that inhibiting IGFIR in hormone-resistant ER-positive cells strongly inhibits their growth (46). Increased PI3K/AKT/mTOR signaling through IGFIR activation is suggested to make breast cancer cells independent of ER signaling for proliferation and survival, conferring endocrine resistance (47). There is also evidence that the IGF/insulin signaling pathway functions differently depending on ER status in breast cancer (48). Mechanisms of endocrine resistance have not been fully elucidated. However, there are inherent differences in gene signaling and upregulation between tamoxifen-resistant cells and aromatase inhibitor-resistant cells, which might explain differences in the prognostic impact of IGFBP7^high^ depending on the type of endocrine treatment (49). As other IGFBPs have been linked to anti-proliferative effects in ER^+^ tumors (48,50), it is possible that IGFBP7 might have similar effects, given their intercorrelation.

In conclusion, tumor-specific IGFBP7^low^ was associated with a good prognosis in breast cancer. The prognostic impact of IGFBP7^high^ varied according to alcohol consumption, ER status and tamoxifen treatment. The results encourage further study to confirm the results and determine whether IGFBP7^high^ might be a useful tool for selecting patients for tamoxifen treatment.

## Supplementary Material

bgab090_suppl_Supplementary_Figure_S1

bgab090_suppl_Supplementary_Figure_S2

bgab090_suppl_Supplementary_Figure_S3

## Abbreviations

AGM: angiomodulin
ANOVA: analysis of variance
BCFI: breast cancer-free interval
BMI: body mass index
CI: confidence interval
Cm: centimeter
DMFI: distant metastasis-free interval
EMT: epithelial-mesenchymal transition
ER: estrogen receptor
H300: Histo 300
HER2: human epidermal growth factor receptor 2
HR: hazard ratio
HR_adj_: adjusted hazard ratio
kDa: kilo Dalton
Kg: kilogram
Ki67: Kiel 67 proliferation factor
IGF: insulin-like growth factor
IGFBP: insulin-like growth factor binding protein
IGFBP3: insulin-like growth factor binding protein 3
IGFBP7: insulin-like growth factor binding protein 7
IGFIR: insulin-like growth factor 1 receptor
IHC: immunohistochemistry
IQR: interquartile range
m: meter
MHT: menopausal hormone therapy
ml: milliliter
mRNA: messenger ribonucleic acid
NoE: number of events
OS: overall survival
PI3K: phosphatidylinositol 3-kinase
PR: progesterone receptor
PSF: prostacyclin-stimulating factor
REMARK: reporting recommendations for tumor marker prognostic study
shRNA: short hairpin ribonucleic acid
TAF: tumor adhesion factor
TCGA: The Cancer Genome Atlas
TMA: tissue microarray
TNBC: triple negative breast cancer
WHR: waist-to-hip ratio
