# Supplementary figures and images for: Prognostic impact of tumor-specific insulin-like growth factor binding protein 7 (IGFBP7) levels in breast cancer: a prospective cohort study
Source: Carcinogenesis. 2021 Oct 4;42(11):1314–25. doi: 10.1093/carcin/bgab090 (PMC8598394; doi:10.1093/carcin/bgab090)

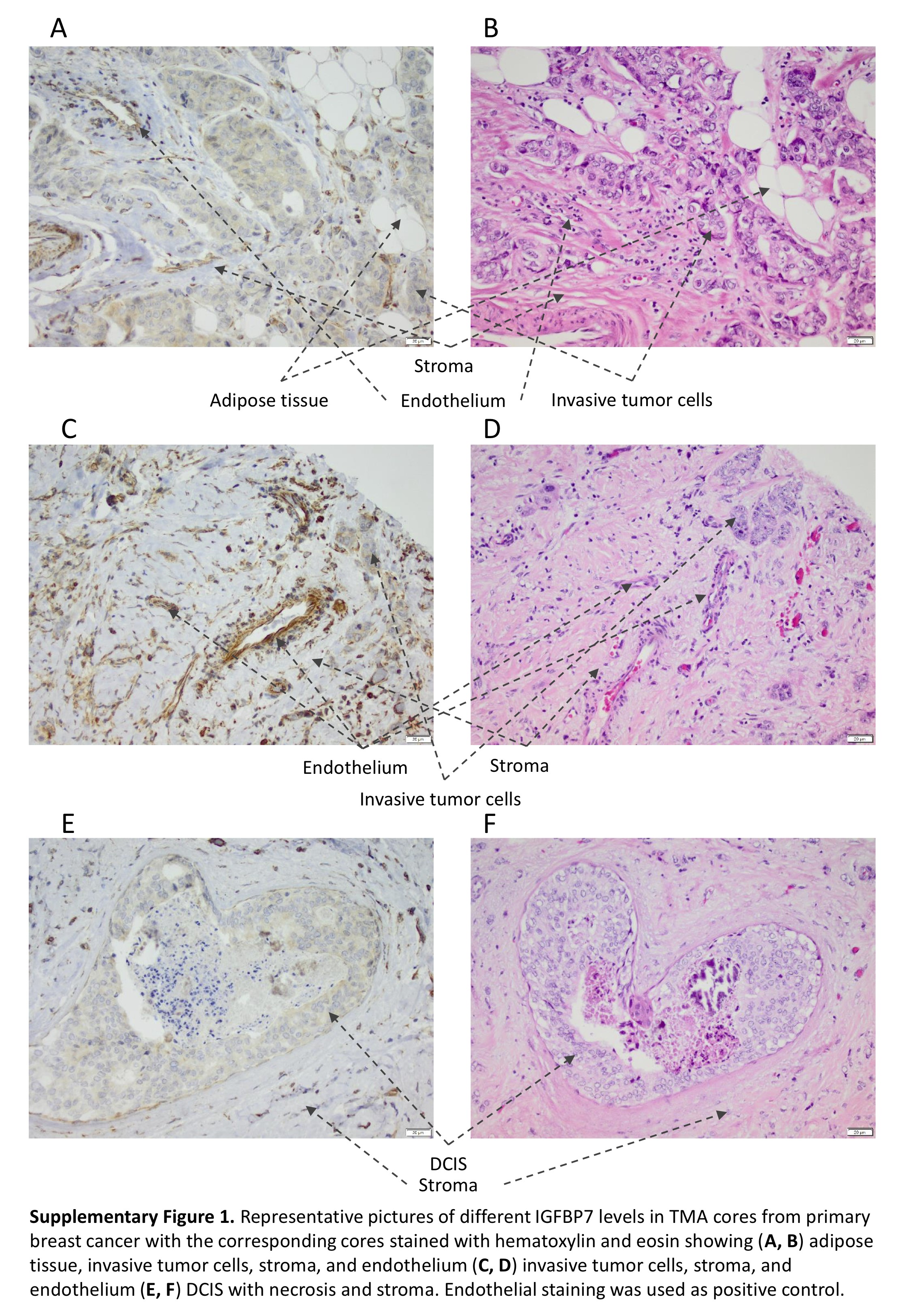

Supplement: bgab090_suppl_Supplementary_Figure_S1 [file bgab090_suppl_supplementary_figure_s1.jpeg]

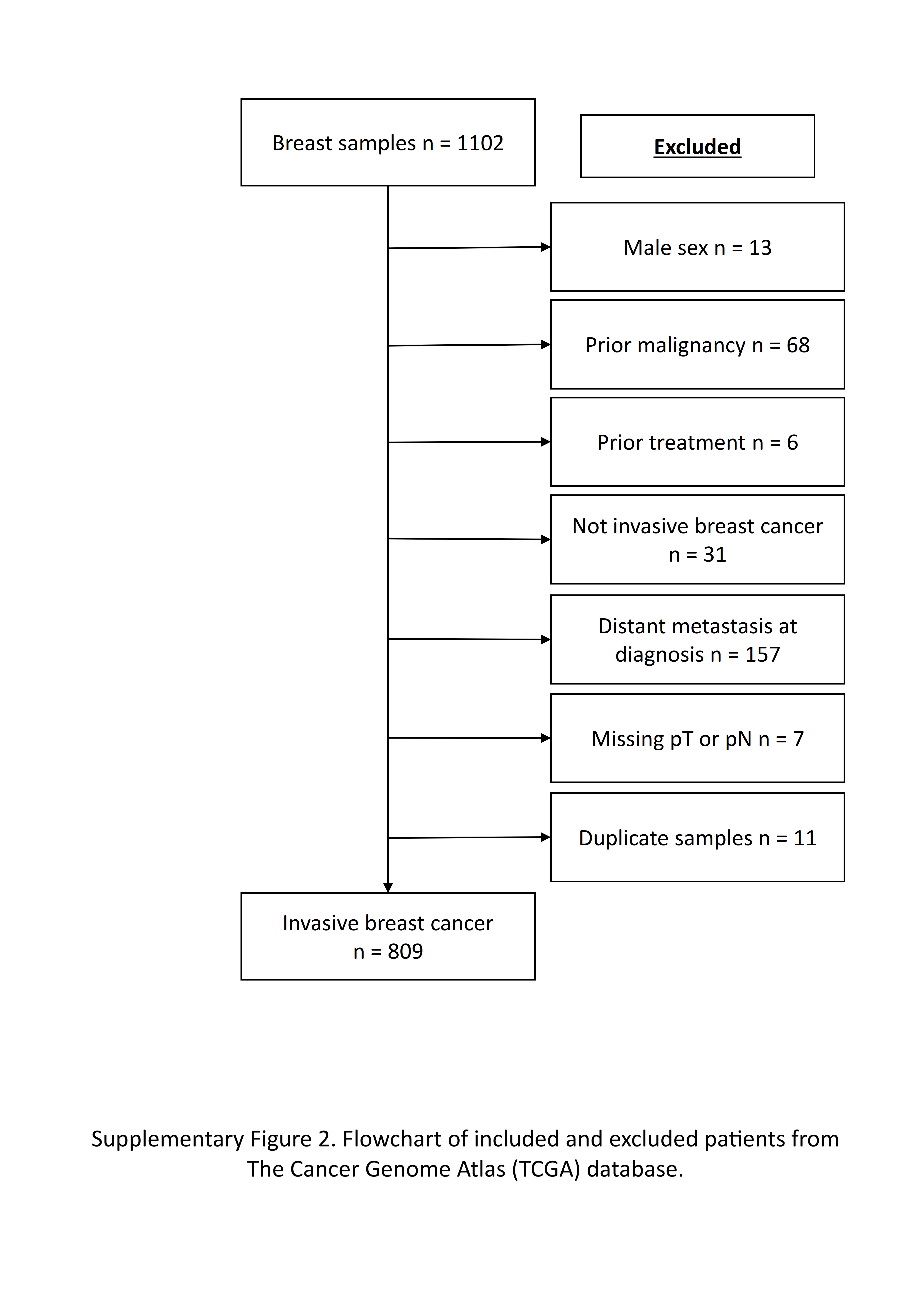

Supplement: bgab090_suppl_Supplementary_Figure_S2 [file bgab090_suppl_supplementary_figure_s2.jpeg]

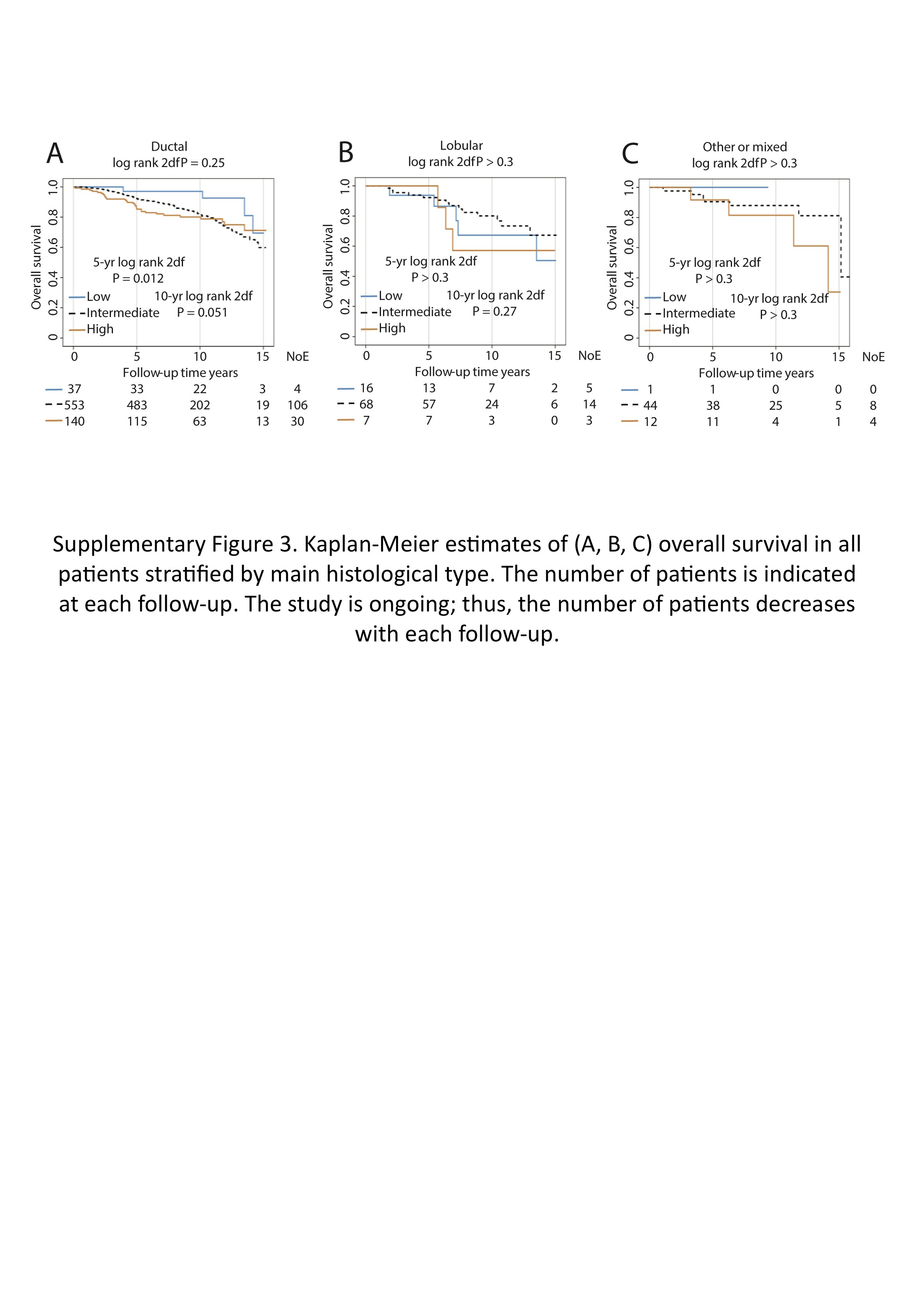

Supplement: bgab090_suppl_Supplementary_Figure_S3 [file bgab090_suppl_supplementary_figure_s3.jpeg]
